# Substrate stiffness influences phenotype and function of human antigen-presenting dendritic cells

**DOI:** 10.1038/s41598-017-17787-z

**Published:** 2017-12-13

**Authors:** Svenja F. B. Mennens, Matteo Bolomini-Vittori, Jorieke Weiden, Ben Joosten, Alessandra Cambi, Koen van den Dries

**Affiliations:** 1 Department of Cell Biology, Radboud Institute for Molecular Life Sciences, Radboud University Medical Center, Geert Grooteplein Zuid 26-28, 6525 GA Nijmegen, The Netherlands; 2 Department of Tumor Immunology, Radboud Institute for Molecular Life Sciences, Radboud University Medical Center, Geert Grooteplein Zuid 26-28, 6525 GA Nijmegen, The Netherlands

## Abstract

Dendritic cells (DCs) are specialized immune cells that scan peripheral tissues for foreign material or aberrant cells and, upon recognition of such danger signals, travel to lymph nodes to activate T cells and evoke an immune response. For this, DCs travel large distances through the body, encountering a variety of microenvironments with different mechanical properties such as tissue stiffness. While immune-related pathological conditions such as fibrosis or cancer are associated with tissue stiffening, the role of tissue stiffness in regulating key functions of DCs has not been studied yet. Here, we investigated the effect of substrate stiffness on the phenotype and function of DCs by conditioning DCs on polyacrylamide substrates of 2, 12 and 50 kPa. Interestingly, we found that C-type lectin expression on immature DCs (iDCs) is regulated by substrate stiffness, resulting in differential antigen internalization. Furthermore, we show that substrate stiffness affects β_2_ integrin expression and podosome formation by iDCs. Finally, we demonstrate that substrate stiffness influences CD83 and CCR7 expression on mature DCs, the latter leading to altered chemokine-directed migration. Together, our results indicate that DC phenotype and function are affected by substrate stiffness, suggesting that tissue stiffness is an important determinant for modulating immune responses.

## Introduction

Dendritic cells (DCs) are key regulators of both the innate and adaptive arms of the immune system. They are considered the most potent antigen-presenting cells and, as such, are the main orchestrators of adaptive immune responses against invading pathogens or aberrant cells. The potential of these cells to control immune responses is well recognized and exploited in anti-cancer immunotherapies where autologous DCs are loaded with tumour antigens to instruct T cells to eradicate tumour cells. This therapeutic approach has been applied already for multiple cancer types, such as melanoma^1–3^, colon cancer^4,5^ and acute myeloid leukaemia^6^. Identifying factors that influence DC phenotype and function will therefore further our understanding of the mechanisms that control immune cell activation and potentially lead to improved DC-based anti-cancer immunotherapies.

DCs undergo a complex differentiation and maturation process during which they drastically change phenotype and function. Immature DCs (iDCs) scan peripheral tissues for intruding pathogens or nascent tumour cells, for which they are equipped with a broad repertoire of pattern recognition receptors (PRRs) such as the mannose receptor (MMR) and DC-SIGN, both members of the class of C-type lectin receptors (CLRs)^7^, which recognize foreign sugar moieties. In addition, iDCs slowly migrate through the extracellular matrix using integrin-based adhesion structures such as focal adhesions and podosomes^8^. Upon antigen recognition and internalization, iDCs mature and acquire a fast migratory phenotype to reach draining lymph nodes^9,10^. This directed migration of mature DCs (mDCs) towards the lymph node is facilitated by a concentration gradient of the chemokines CCL19 and CCL21, sensed through the chemokine receptor CCR7, which is highly expressed on the membrane of mDCs^11^. In addition, mDCs have a high expression of MHC molecules and co-stimulatory molecules such as CD86 and CD83, facilitating antigen presentation and T cell activation to clear pathogens or tumour cells from the body^9,12^. Importantly, while a lot is known on the effect of biochemical signals such as cytokines and chemokines on these key aspects of DC biology, not much is known on the role of mechanical signals on DC phenotype and function.

Since DCs are present in many tissues throughout the body during their lifespan, they encounter many different microenvironments. It is likely that DC function is not only affected by biochemical factors, but also by mechanical stimuli such as shear flow in blood and lymph vessels, stretch and compression in the skin or the lungs, and large stiffness variations throughout the different tissues. Tissue stiffness is defined as the resistance of a tissue to deformation and ranges from ~0.2 kPa in the lungs to ~15 kPa in skeletal muscle or cartilage^13,14^. Tissue stiffness is known to affect mesenchymal stem cell differentiation^15^, fibroblast migration^16^, neuron morphology and branching^17^, and endothelial cell and fibroblast adhesion^18^. Importantly, during immune-related pathological conditions such as fibrosis^19^ or tumour progression^20^, tissue stiffness is known to change. It is therefore particularly interesting that tissue stiffness has been shown to also influence cellular responses in a large diversity of immune cells such as macrophages^21–23^, neutrophils^24^, T cells^25^ and B cells^26^. Yet, the role of tissue stiffness in regulating the key functions of iDCs and mDCs has not been investigated yet.

In this study, we conditioned human monocyte-derived DCs (moDCs), a well-established and frequently used model for DCs, on substrates with different stiffness (2, 12 and 50 kPa) and studied the effect on several key functions of iDCs and mDCs. Our results indicate that CLR expression by iDCs is regulated by substrate stiffness, resulting in differential internalization of CLR-binding antigens. Furthermore, we show that substrate stiffness affects the expression of β_2_ integrins and podosome formation by iDCs. Finally, we demonstrate that substrate stiffness influences CD83 and CCR7 expression on mDCs, the latter leading to altered chemokine-directed migration. Together, these results indicate that DCs can sense substrate stiffness during differentiation and maturation, leading to alterations in both iDC and mDC phenotype that can critically affect their function and eventual *in vivo* application.

## Results

### Substrate stiffness does not greatly influence iDC spreading behaviour

**Figure 1:**
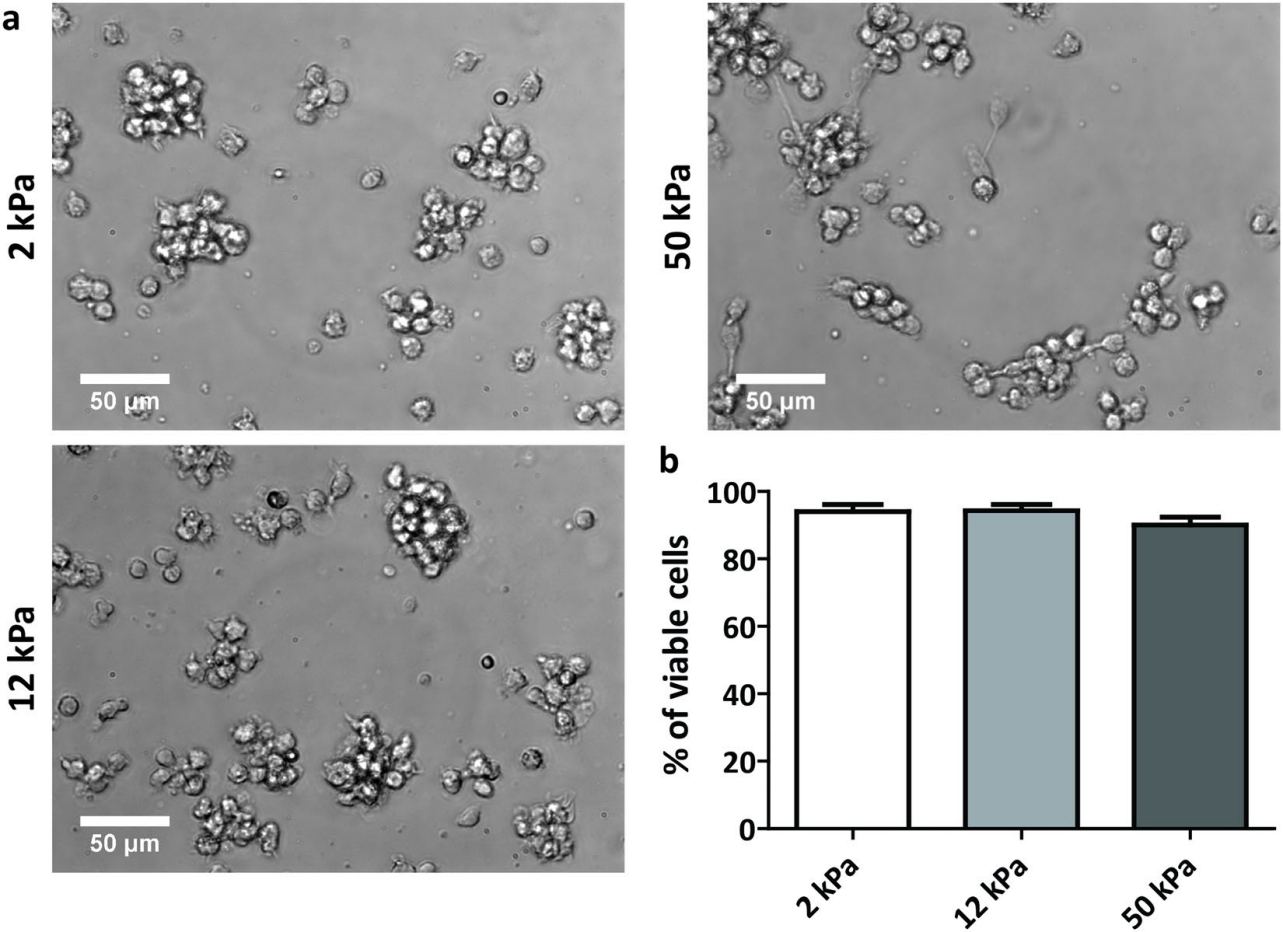
Spreading behaviour and viability of iDCs are not affected by substrate stiffness. (**a**) Representative brightfield images taken with a 40x objective of iDCs in culture on PAA substrates of 2, 12 or 50 kPa after 3 days of differentiation. (**b**) Viability of iDCs after 6 days of differentiation on PAA substrates of 2, 12, or 50 kPa (n = 6 donors), represented as the eFluor780-negative fraction of cell population measured by flow cytometry. Bars represent mean with SEM.

To investigate the effect of substrate stiffness on DC phenotype and functionality, we used commercially available activated polyacrylamide (PAA) substrates with stiffness values of 2, 12 and 50 kPa, reflecting the range of stiffness values DCs possibly encounter *in vivo*. Before cell seeding, the PAA substrates were pretreated with medium containing 2% human serum to occupy the reactive groups of the activated PAA while providing ligands to promote cell adhesion. Upon seeding, we noticed that the monocytes adhered similarly to all substrates. Since substrate stiffness is known to influence cell spreading^18^, we examined the morphology of iDCs at day 3 of differentiation using brightfield imaging of the cell cultures (Fig. 1a and Supplementary Fig. S1). Interestingly, no stiffness-dependent differences in cell spreading behaviour were observed for the 2, 12, 50 kPa PAA substrates. iDCs on all PAA substrates were rounded and tended to engage in cell-cell adhesion, thereby forming small cell clumps that adhered weakly to the underlying surface (Fig. 1a). This spreading behaviour is remarkably different from day 3 iDCs on standard tissue culture plastic, which were strongly adherent and well spread (Supplementary Fig. S1), as we and others have shown before. Together, these results indicate that, while iDC spreading is controlled by the type of substrate, substrate stiffness does not greatly influence the spreading behaviour of iDCs in the stiffness range tested.

### iDCs conditioned on PAA are viable and negative for monocyte or macrophage markers

Next, to evaluate the survival of iDCs on the different PAA substrates, we determined DC viability after 6 days of differentiation (Fig. 1b). No major changes in viability were detected, with less than 10% of average cell death in all conditions (Fig. 1b), indicating that the PAA substrates were not toxic to the cells over longer culturing periods. Furthermore, we also tested whether the iDCs conditioned on the various PAA substrates lost their monocyte characteristics and did not acquire a macrophage phenotype by determining the expression of CD14 and CD68 by flow cytometry, respectively (Supplementary Fig. S2). For this experiment, iDCs conditioned on tissue culture plastic were taken along to verify iDC phenotype under standard culture conditions. These data clearly show that the iDCs conditioned on 2, 12 and 50 kPa were negative for CD14 as well as CD68, indicating that the cells differentiated upon the addition of IL-4 and GM-CSF and most likely acquired a DC-like phenotype.

### Substrate stiffness regulates C-type lectin expression on iDCs

**Figure 2:**
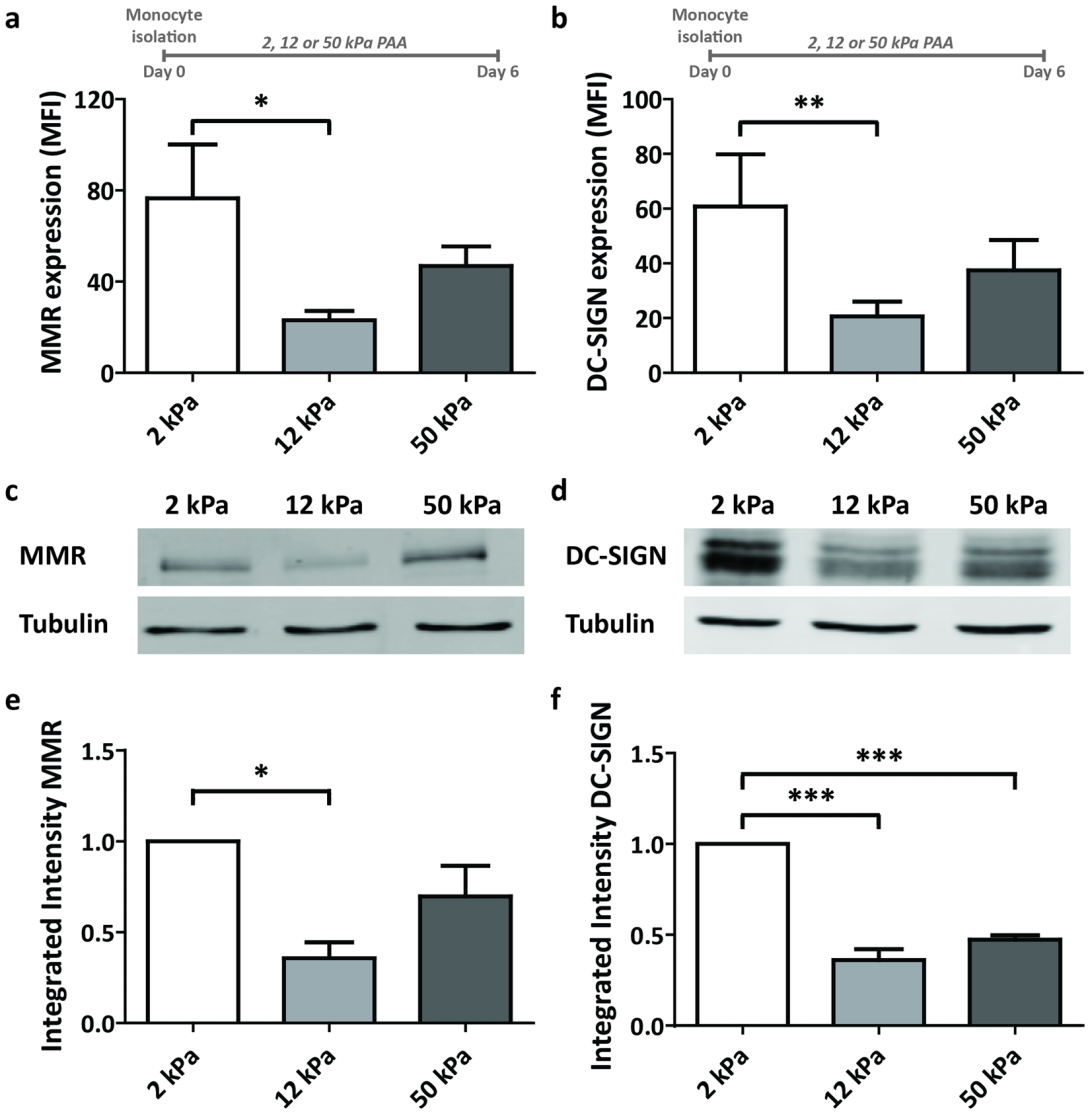
Substrate stiffness regulates C-type lectin expression on iDCs. (**a**,**b**) Cell surface expression of C-type lectins MMR (**a**, n = 7 donors) and DC-SIGN (**b**, n = 7 donors) on iDCs conditioned for 6 days on PAA substrates of 2, 12 and 50 kPa, represented as geometric mean fluorescence intensity measured with flow cytometry. Bars represent mean with SEM. Statistical significance was tested with repeated measures ANOVA. (**c**,**d**) Representative images of western blot analysis of total MMR protein expression (**c**) and total DC-SIGN protein expression (**d**) in iDCs conditioned for 6 days on PAA substrates of 2, 12 or 50 kPa. Unprocessed original scans of blots are shown in Supplementary Fig. S9. (**e**,**f**) Quantification of integrated intensity of total MMR protein expression (**e**, n = 3 donors) and total DC-SIGN protein expression (**f**, n = 3 donors) measured by Western Blot analysis in iDCs conditioned for 6 days on PAA substrates of 2, 12 or 50 kPa. Per donor, integrated intensities are normalized to 2 kPa results. Bars represent mean with SEM. Statistical significance was tested with repeated measures ANOVA. *p < 0.05; ***p < 0.001.

Pathogen recognition and uptake by C-type lectin receptors (CLRs) is a hallmark function of iDCs^27^. To test whether substrate stiffness influences the upregulation of CLRs during DC differentiation, we determined the surface expression of the CLRs MMR (Macrophage Mannose Receptor, CD206)^28^ and DC-SIGN^29^ (Dendritic-Cell Specific Intercellular adhesion molecule-3-Grabbing Non-integrin, CD209) on day 6 iDCs conditioned on 2, 12 and 50 kPa by flow cytometry. Interestingly, we found that the MMR and DC-SIGN expression was more than 3 fold lower on iDCs conditioned on 12 kPa compared to DCs conditioned on 2 kPa (Fig. 2a,b). Expression of both CLRs was intermediate on iDCs conditioned on 50 kPa, not being significantly different from the expression on iDCs conditioned on 2 and 12 kPa (Fig. 2a,b). To note, conditioning iDCs on PAA substrates of 0.5 kPa did not further increase the expression of MMR and DC-SIGN compared to 2 kPa (Supplementary Fig. S3), suggesting that C-type lectin expression in iDCs is not influenced by stiffness values lower than 2 kPa. To test whether the differences observed for 2, 12 and 50 kPa by flow cytometry were due to different receptor recycling dynamics or global receptor expression, we prepared whole cell lysates from iDCs conditioned on 2, 12 and 50 kPa and performed a western blot for MMR and DC-SIGN (Fig. 2c–f). Interestingly, the amount of MMR and DC-SIGN in the whole cell lysates correlated directly with the results from the flow cytometry, indicating that the observed differences are due to global receptor expression.

To exclude that the differences in CLR expression were induced by a differential ligand coating between 2, 12, and 50 kPa PAA substrates, we conditioned iDCs on substrates pretreated either with medium containing 2% human serum (serum), fibronectin (FN), or with FN followed by medium containing 2% human serum (FN + serum). First, to evaluate the coating efficiency of proteins on the 2, 12 and 50 kPa PAA substrates, we fluorescently labelled the FN-coating with an anti-FN antibody and evaluated the staining by widefield microscopy (Supplementary Fig. S4). These results demonstrated that the mean fluorescence intensity does not depend on PAA stiffness or on the FN concentration, indicating that the coating efficiency of proteins on the various substrates is similar. Importantly, we detected no substantial differences in DC-SIGN expression among the coating conditions (Supplementary Fig. S4), indicating that the surface coating does not influence the expression of DC-SIGN and strongly suggesting that the differential CLR expression of day 6 iDCs conditioned on the various PAA substrates is dependent on substrate stiffness.

To investigate whether an initial increase in the expression of MMR and DC-SIGN in iDCs could be reversed by exposing the iDCs to 12 kPa PAA during the last days of differentiation, we cultured monocytes for 3 days on standard tissue culture plastic (Day 0–3) and then transferred them to 12 kPa PAA and cultured them for 3 more days (Day 3–6) (Supplementary Fig. S5). Cells that were transferred and cultured for 3 more days in fresh tissue culture plates were taken along for comparison. Importantly, for this experiment, we used standard tissue culture plastic to induce the expression of MMR and DC-SIGN, since the transfer of cells between PAA substrates would increase the technical variability of this experimental setup considerably. As expected, after 3 days in standard tissue culture flasks, iDCs expressed both MMR and DC-SIGN (Supplementary Fig. S5). Strikingly, when seeded for 3 more days on 12 kPa, MMR and DC-SIGN expression decreased 3-4 fold, whereas their expression increased further or remained stable after 3 more days on plastic. This indicates that 12 kPa PAA substrates present a strong inhibitory signal for CLR expression in iDCs, even when CLRs are already expressed. Furthermore, since IL-4 is known to be the primary cytokine to induce CLR expression in iDCs^30^, we investigated whether CLR expression could be rescued by adding double amounts of IL-4 to the culture medium when cells were transferred at day 3 (Supplementary Fig. S6). Interestingly, we found that adding more IL-4 to the culture medium did not result in an increase in MMR or DC-SIGN expression, suggesting that the substrate properties are dominant over the cytokine signalling in regulating CLRs.

Altogether, these results demonstrate that stiffness values of 12 kPa inhibit the expression levels of MMR and DC-SIGN, regardless of ligand coating, differentiation stage and IL-4 concentration and strongly suggest that tissue stiffness controls CLR expression in iDCs.

### iDCs conditioned on substrates with varying stiffness display differential C-type lectin-dependent antigen internalization

**Figure 3:**
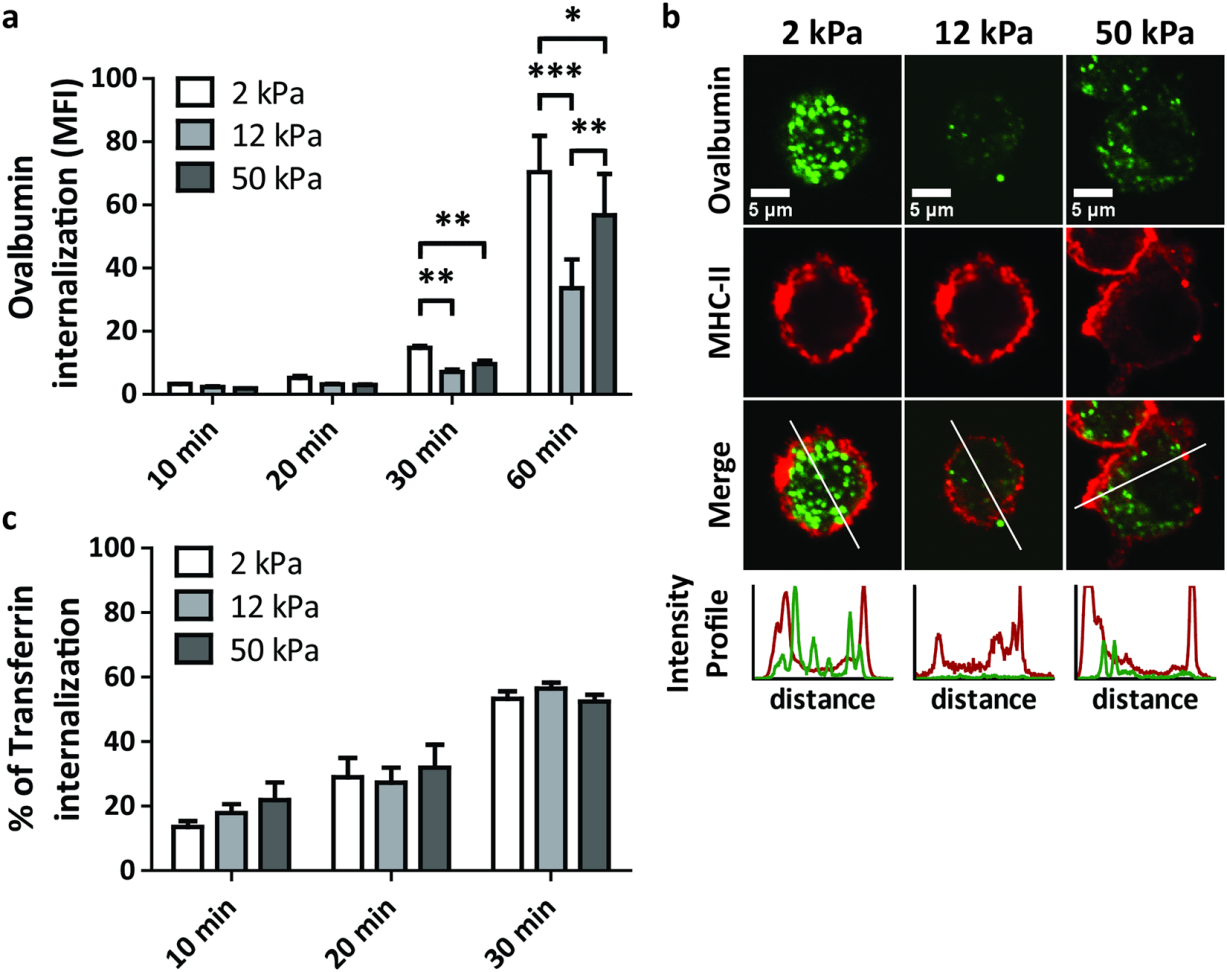
iDCs conditioned on substrates with varying stiffness display differential CLR-dependent antigen internalization. (**a**) Time course of internalization of ovalbumin-Alexa488 by iDCs conditioned for 6 days on PAA substrates of 2, 12, or 50 kPa (n = 3 donors), represented by geometric mean fluorescence intensity measured with flow cytometry. Bars represent mean with SEM. Statistical significance was tested per time point with repeated measures ANOVA. (**b**) Representative confocal images of day 6 iDCs after 60 minutes of internalization of ovalbumin-Alexa488. Extracellular MHC-II staining indicates the outline of the cell. Representative intensity profiles of Ovalbumin-Alexa488 and MHC-II are shown to indicate the intracellular localization of Ovalbumin-Alexa488. (**c**) Time course of transferrin internalization by iDCs conditioned for 6 days on PAA substrates of 2, 12, or 50 kPa (n = 3 donors), represented as % of internalization, based on geometric mean fluorescence intensity measured with flow cytometry. Bars represent mean with SEM. Statistical significance was tested per time point with repeated measures ANOVA. *p < 0.05; **p < 0.01; ***p < 0.001.

Since CLRs are important for antigen uptake by iDCs, we expected the stiffness-dependent differences in DC-SIGN and MMR surface expression to influence antigen binding and internalization. We therefore exposed iDCs which were conditioned on 2, 12, and 50 kPa to Alexa488-conjugated ovalbumin, an antigen internalized predominantly via MMR^31^, and determined its internalization by flow cytometry and confocal microscopy (Fig. 3a,b). We observed that, after 30 minutes, iDCs conditioned on 2 kPa had taken up 1.5–2 fold more ovalbumin compared to iDCs conditioned on 12 or 50 kPa and that, after 60 minutes, these iDCs continued to be the most effective in taking up ovalbumin (Fig. 3a). Furthermore, although iDCs conditioned on 12 and 50 kPa were equally capable of taking up ovalbumin after 30 minutes, iDCs from 50 kPa had taken up significantly more (>1.5 fold) ovalbumin than the iDCs from 12 kPa after 60 minutes (Fig. 3a), indicating that the ability of iDCs to take up ovalbumin correlated positively with the observed MMR expression (Fig. 2a). To confirm that the ovalbumin detected by flow cytometry is indeed internalized, and not only sticking to the iDCs, confocal images were taken of iDCs that were exposed for 60 minutes to Alexa488-conjugated ovalbumin (Fig. 3b). These images clearly demonstrated that the ovalbumin was taken up by the iDCs. Importantly, we found no differences in transferrin internalization among the differentially conditioned iDCs (Fig. 3c), indicating that the differences in ovalbumin internalization are not the result of a general impact on all endocytic processes. Together, these results indicate that the differential MMR expression induced by substrate stiffness has important functional consequences for iDCs, and suggests that tissue stiffness affects the ability of iDCs to bind and internalize antigens.

### Substrate stiffness influences β_2_ integrin expression and podosome formation in iDCs

**Figure 4:**
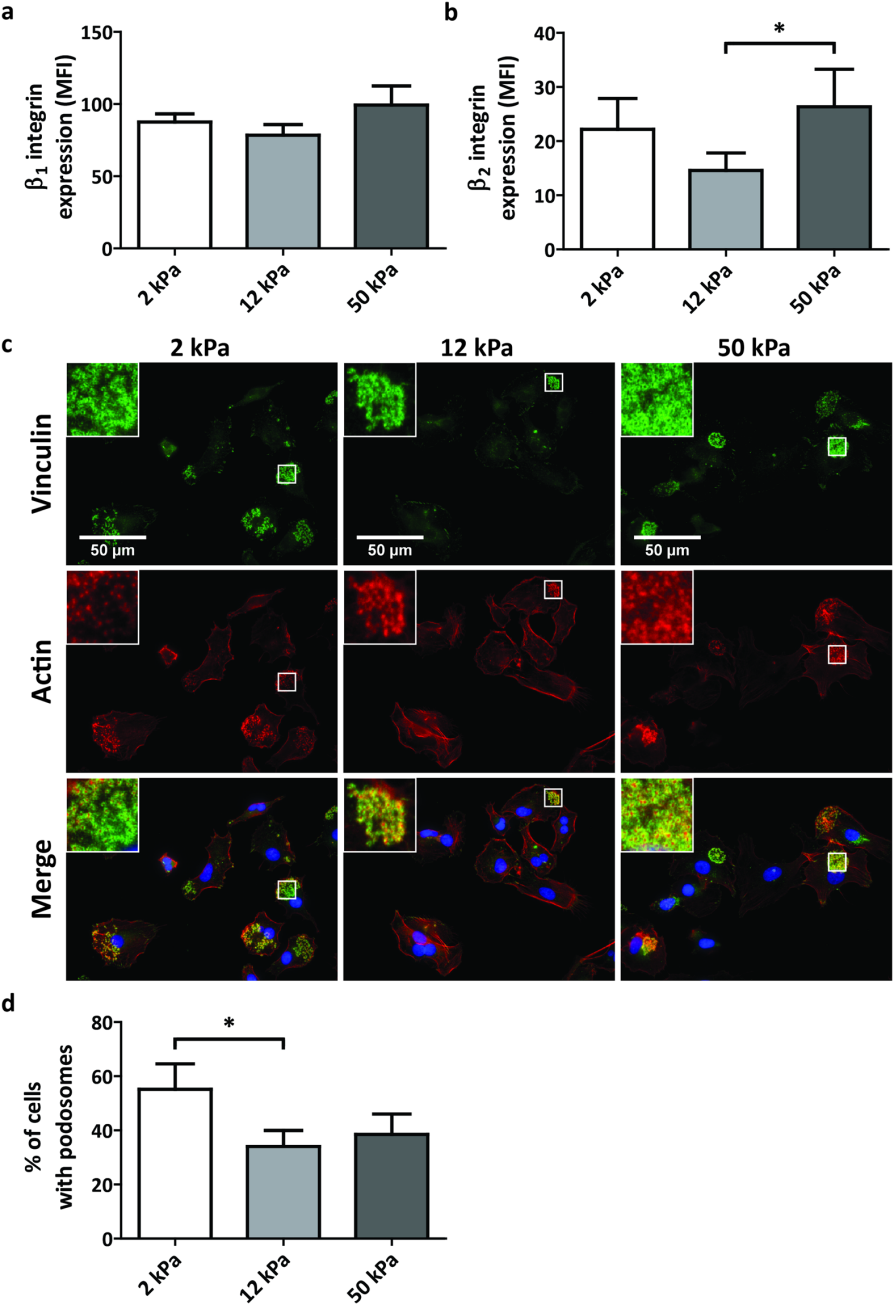
Substrate stiffness influences β_2_ integrin expression and podosome formation in iDCs. (**a,b**) Cell surface expression of total β_1_ integrins (**a**, n = 6 donors) and total β_2_ integrins (**b**, n = 8 donors) from iDCs conditioned for 6 days on PAA substrates of 2, 12 or 50 kPa, represented as geometric mean fluorescence intensity measured with flow cytometry. Bars represent mean with SEM. Statistical significance was tested with repeated measures ANOVA. (**c**) Representative widefield images of iDCs conditioned for 6 days on PAA substrates of 2, 12 or 50 kPa and seeded for 3 hours on FN-coated glass. Shown are vinculin (green), actin (red), and the merged image that includes the nucleus (blue). Inserts top left show enlargement of podosomes, indicated in the white square. (**d**) Fraction of cells forming podosomes from iDCs conditioned for 6 days on PAA substrates of 2, 12 or 50 kPa (n = 4 donors). More than 50 cells per condition were analysed and only cells containing clear actin dots, accompanied by vinculin rings were counted as positive for podosomes. Bars represent mean with SEM. Statistical significance was tested with repeated measures ANOVA. *p < 0.05.

To scan peripheral tissues for pathogens and aberrant cells, iDCs slowly migrate through peripheral tissue in an integrin-dependent manner. To evaluate the role of substrate stiffness in regulating integrin expression in iDCs, we determined the expression of total β_1_ and β_2_ integrins on the surface of iDCs conditioned on 2, 12 and 50 kPa by flow cytometry. Interestingly, while we did not find significant differences in β_1_ integrin surface expression among the different PAA substrates (Fig. 4a), we did find stiffness-dependent changes for β_2_ integrins. Expression of total β_2_ integrins was 1.5–2 fold lower on iDCs conditioned on 12 kPa compared to iDCs conditioned on 2 or 50 kPa (Fig. 4b). To evaluate whether even softer stiffness values would lead to a further increase in the expression of β_2_ integrins, we conditioned iDCs on PAA substrates of 0.5 kPa and evaluated its cell surface expression by flow cytometry (Supplementary Fig. S7). Interestingly, we did not observe significant differences in β_2_ integrin expression between 0.5 kPa and 2 kPa.

In iDCs, β_2_ integrins are important for the formation of circular adhesion structures called podosomes^32^, which are characterized by an actin-rich core surrounded by a vinculin ring. To test the ability of iDCs conditioned on 2, 12 and 50 kPa to form podosomes, we seeded these cells on FN-coated glass coverslips and evaluated podosome formation with widefield fluorescence microscopy by staining cells for vinculin and actin (Fig. 4c,d). Cells containing at least 5 podosomes were scored as podosome-positive (Fig. 4c, Image insets). Interestingly, we observed that cells obtained from all substrates were able to spread on glass, but iDCs conditioned on 12 kPa showed a significantly lower percentage of podosome forming cells than iDCs conditioned on 2 kPa (Fig. 4d). Altogether, these results indicate that conditioning iDCs on substrates with different stiffness influences their expression of β_2_, but not β_1_ integrins as well as their ability to assemble podosomes and suggest that tissue stiffness controls iDC adhesive and migratory behaviour.

### Substrate stiffness affects mDC adhesive behaviour and viability, but not motility

**Figure 5:**
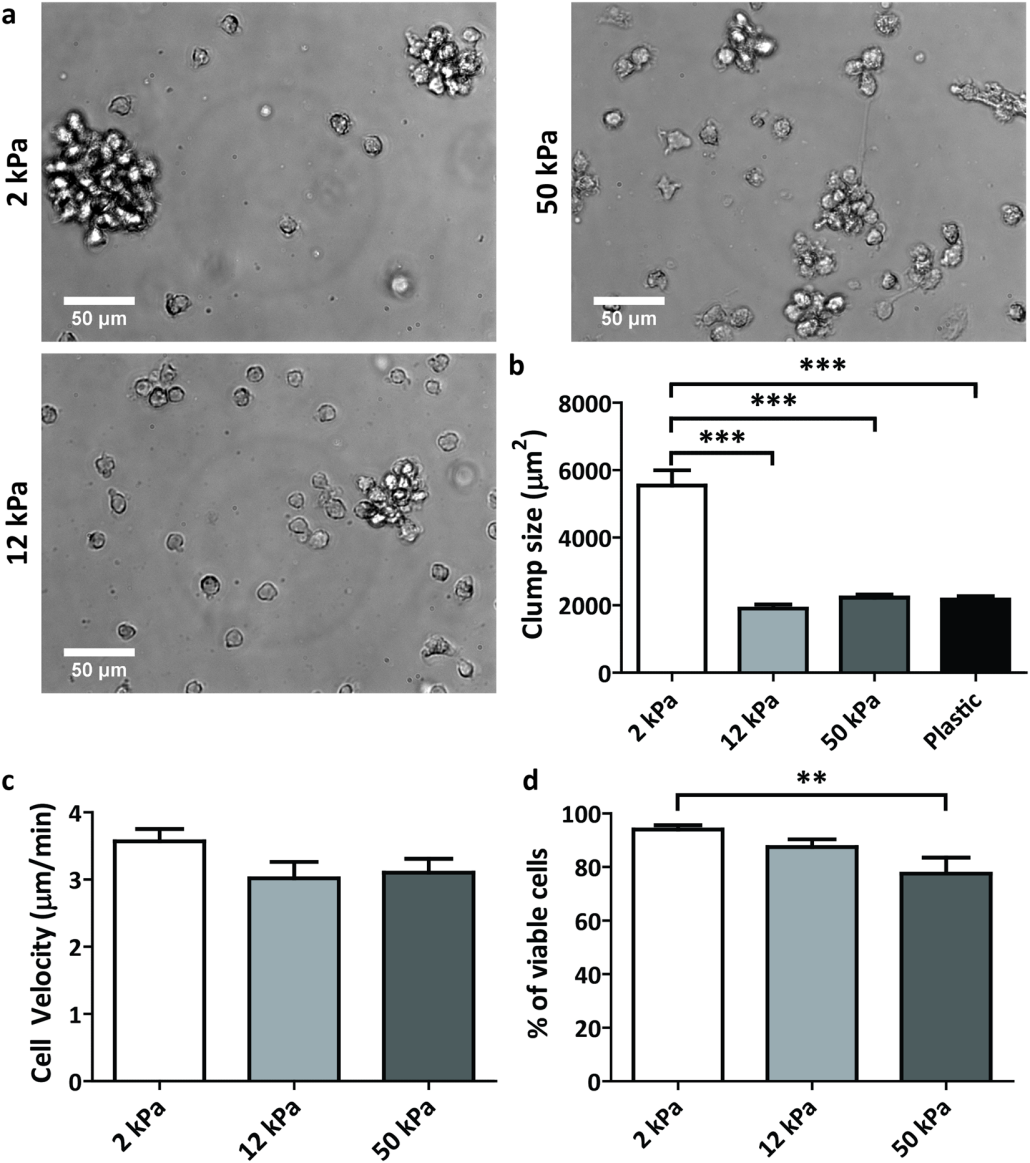
Substrate stiffness affects mDC adhesive behaviour and viability, but not motility. (**a**) Representative brightfield images taken with a 40x objective of mDCs in culture on PAA substrates with stiffness of 2, 12 or 50 kPa after 1 day of maturation by addition of IL-4, GM-CSF, IL-1β, TNF-α, IL-6 and PGE_2_ (total 5 days of culture). (**b**) Cell clump size (in µm^2^) in mDC cell culture on PAA substrates with stiffness of 2, 12 or 50 kPa after 1 day of maturation (total 5 days of culture). More than 40 clumps in more than 8 fields of view per condition, were measured in brightfield images of 2 donors using in FIJI ImageJ. Bars represent mean with SEM. Statistical significance was tested with one-way ANOVA. (**c**) Cell velocity (in µm/min) of mDCs on PAA substrates with stiffness of 2, 12 or 50 kPa after 1 day of maturation (total 5 days of culture). Cell velocity was determined by manually tracking >30 single cells per condition over 1 hour time period in brightfield images of 2 donors, using the Manual Tracking plugin in FIJI ImageJ. Bars represent mean with SEM. (**d**) Viability of day 2 mDCs (n = 8 donors) conditioned on PAA substrates of 2, 12, or 50 kPa, represented as the eFluor780-negative fraction of cell population measured with flow cytometry. Bars represent mean with SEM. Statistical significance was tested with repeated measures ANOVA. **p < 0.01; ***p < 0.001.

Upon antigen recognition and uptake, iDCs mature and migrate to lymph nodes to stimulate T cells. To investigate the effects of substrate stiffness on DC maturation, monocytes were first differentiated on the 2, 12, and 50 kPa PAA substrates for 4 days in the presence of IL-4 and GM-CSF (day 4 iDCs), followed by 2 more days of maturation in the presence of the pro-inflammatory cocktail IL-4, GM-CSF, IL-1β, TNF-α, IL-6 and PGE_2_ (day 2 mDCs). We first examined the adhesive behaviour of mDCs at day 1 of maturation using brightfield microscopy (Fig. 5a). We observed that mDCs on the various PAA substrates round up even more than during differentiation and that a large percentage detaches from the substrate (Fig. 5a), something which is also observed when cells are seeded on tissue culture plastic (Supplementary Fig. S8). Furthermore, on 2 kPa, cell-cell adhesion was significantly increased after maturation compared to 12 and 50 kPa and tissue culture plastic, since large clumps of cells were observed in cell culture after one day of maturation (Fig. 5a,b).

One of the hallmarks of DC maturation is the increased motility of mDCs compared to iDCs^10^. To investigate whether substrate stiffness would influence DC motility, we analysed the velocity of day 1 mDCs undergoing 2D random migration on the 2, 12 and 50 kPa PAA substrates (Fig. 5c). We found that mDC velocity is very much comparable to values we have published before^10^ and that there are no significant differences between the various PAA substrates, indicating that substrate stiffness does not influence the motility of mDCs in the stiffness range tested.

To evaluate the survival of mDCs, we determined their viability by flow cytometry after 2 days of maturation. Here, we observed a small, but significant, difference in cell viability for mDCs that were conditioned on 50 kPa as compared to mDCs conditioned on 2 kPa (Fig. 5d). It is important to note that we therefore included a viability staining in the gating strategy for all flow cytometry experiments with day 2 mDCs.

### Substrate stiffness regulates CD83 expression, but not T cell activation capacity, of mDCs

**Figure 6:**
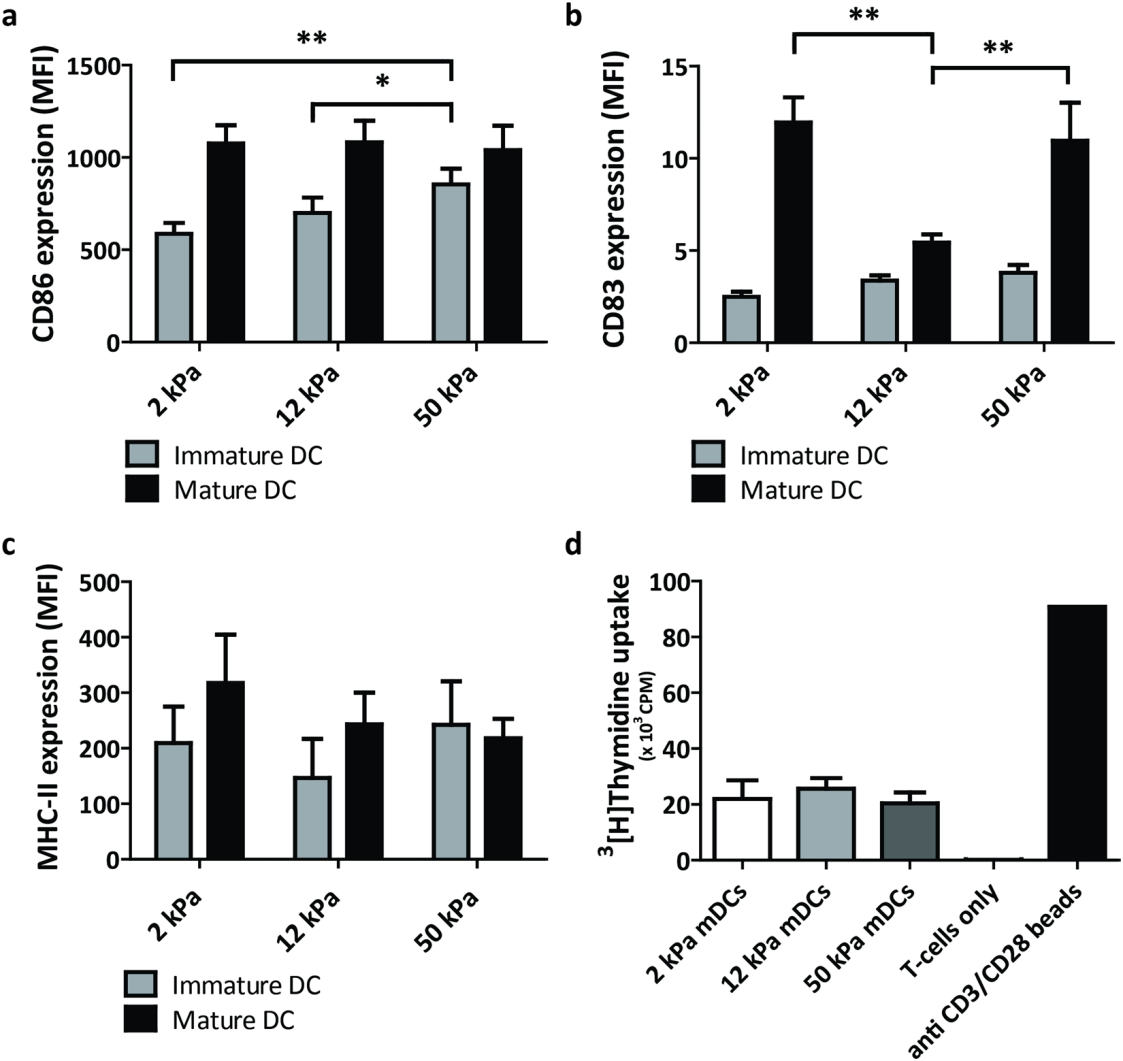
Substrate stiffness regulates CD83 expression, but not T-cell activation capacity, of mDCs. (**a**,**b**,**c**) Cell surface expression of CD86 (**a**, n = 7 donors), CD83 (**b**, n = 7 donors) and MHC-II (n = 5 donors) in day 4 iDCs (gray bars) and day 2 mDCs (black bars) conditioned on PAA substrates of 2, 12 and 50 kPa, represented as geomean fluorescence intensity measured with flow cytometry. Maturation was induced by addition of IL-4, GM-CSF, IL-1β, TNF-α, IL-6 and PGE_2_. Bars represent mean with SEM. Statistical significance among iDC/mDC conditions was tested with repeated measures ANOVA. (**d**) T-cell proliferation of allogeneic PBLs stimulated by day 2 mDCs (n = 2 donors, technical triplicates) conditioned on PAA substrates of 2, 12 or 50 kPa (at a ratio of 10:1), measured with ^3^H-Thymidine incorporation detected in counts per minute. Negative control contained no stimulus (medium only) and positive control contained anti CD3/CD28 T-cell activating beads at a ratio of 4:1. Bars represent mean with SEM. *p < 0.05; **p < 0.01.

T cell stimulation in the lymph nodes is mainly promoted by MHC class II and co-stimulatory molecules such as CD83 and CD86 on the plasma membrane of mDCs. To investigate the role of substrate stiffness in upregulating these maturation markers, we determined their surface expression in both day 4 iDCs and day 2 mDCs with flow cytometry (Fig. 6a–c). For CD86, cell surface expression was already detected at relatively high levels in day 4 iDCs, and was significantly higher on day 4 iDCs conditioned on 50 kPa compared to iDCs conditioned on 2 and 12 kPa (Fig. 6a). After maturation, no significant differences were detected in CD86 expression levels between the day 2 mDCs conditioned on the various PAA substrates. For CD83, cell surface expression was very low in day 4 iDCs, and clearly upregulated on mDCs conditioned on 2 and 50 kPa (Fig. 6b). Interestingly, mDCs conditioned on 12 kPa only displayed a very minor upregulation of CD83, which was significantly lower compared to day 2 mDCs conditioned on 2 and 50 kPa. For MHC-II, we found no significant differences between cells from the different substrates, neither in an immature nor in a mature state (Fig. 6c). Together, these results indicate that the upregulation of CD83 in mDCs is regulated by substrate stiffness and suggest that tissue stiffness may affect the ability of mDCs to stimulate T cells.

To test whether substrate stiffness controls the potential of mDCs to induce T cell proliferation, we performed a mixed lymphocyte reaction (MLR) where allogeneic PBLs were incubated with mDCs conditioned on 2, 12 and 50 kPa (Fig. 6d). Interestingly, T cell proliferation, as measured by thymidine incorporation was similar under all conditions, indicating that conditioning mDCs on the various PAA substrates did not influence their ability to stimulate T cells.

### Substrate stiffness influences chemokine-directed migration of mature DCs

**Figure 7:**
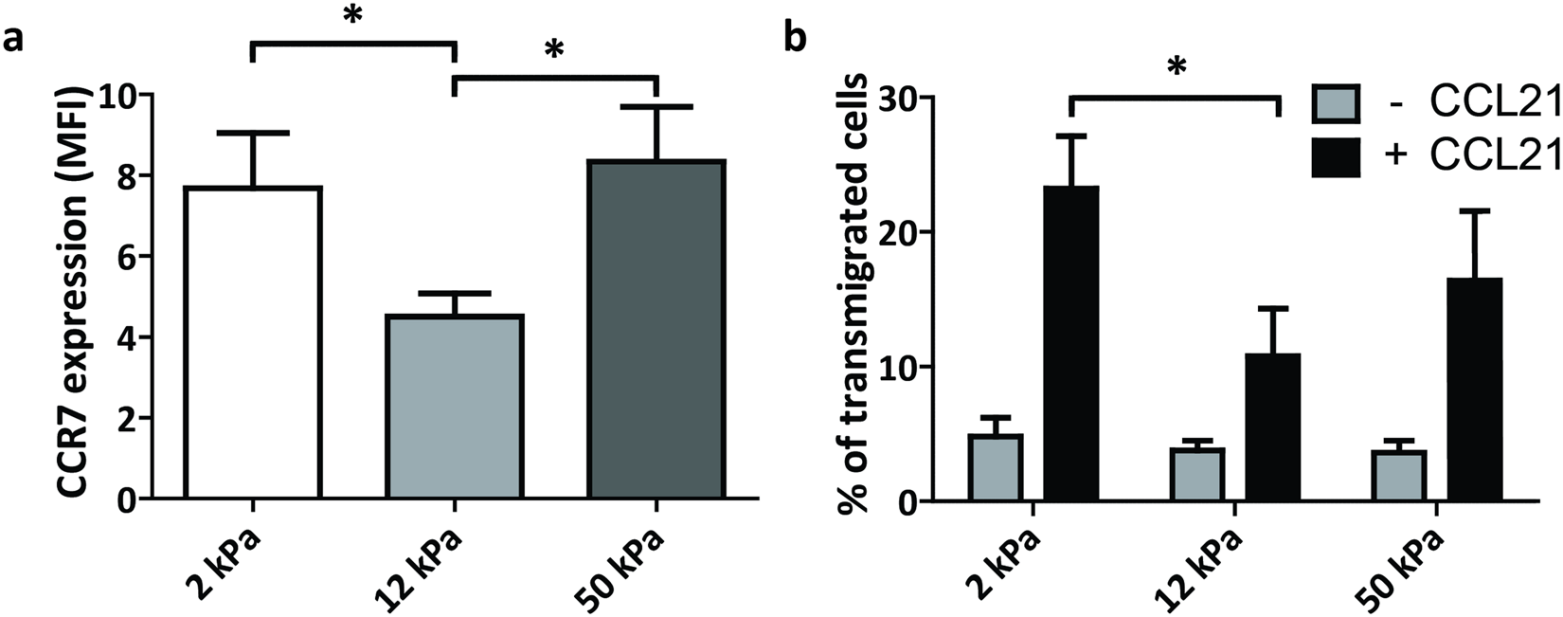
Substrate stiffness influences chemokine-directed migration of mDCs. (**a**) Cell surface expression of CCR7 (n = 7 donors) in day 2 mDCs conditioned on PAA substrates of 2, 12 and 50 kPa, represented as geomean fluorescence intensity measured with flow cytometry. Maturation was induced by addition of IL-4, GM-CSF, IL-1β, TNF-α, IL-6 and PGE_2_. Bars represent mean with SEM. Statistical significance was tested with repeated measures ANOVA. (**b**) Percentage of transmigrated day 2 mDCs displaying random (−CCL21) or chemokine-directed (+CCL21) migration, as determined from cell counts measured by flow cytometry (n = 4 donors, technical duplicates). Maturation was induced by addition of IL-4, GM-CSF, IL-1β, TNF-α, IL-6 and PGE_2_. Bars represent mean with SEM. Statistical significance between conditions with or without CCL21 was tested with repeated measures ANOVA. *p < 0.05.

mDC migration from peripheral tissues to lymph nodes is regulated by the chemokine receptor CCR7 that senses a positive gradient of the chemokines CCL19 and CCL21 towards lymph vessels. To investigate the role of substrate stiffness on mDC responsiveness to chemokines, we first determined the cell surface expression of CCR7 in mDCs conditioned on 2, 12 and 50 kPa. Interestingly, we found that CCR7 was significantly lower in mDCs conditioned on 12 kPa compared to mDCs conditioned on 2 and 50 kPa (Fig. 7a). To investigate the functional consequences of the differential expression of CCR7, we performed a transwell migration assay, allowing mDCs to migrate towards the chemokine CCL21 (Fig. 7b). In the absence of CCL21, less than 10% of the mDCs migrated across the membrane in 2.5 hours and no significant differences were observed among the different conditions. In the presence of CCL21, however, there was a striking difference in chemokine-directed migration. mDCs conditioned on 12 kPa displayed a significantly lower transwell migration compared to mDCs conditioned on 2 kPa substrates, correlating positively with the CCR7 expression in these cells (Fig. 7b). mDCs conditioned on 12 kPa also displayed a lower transwell migration compared to mDCs conditioned on 50 kPa, but these results were not significant. Together, these results indicate that substrate stiffness regulates the expression of CCR7 in mDCs, thereby altering their capacity to migrate towards the chemokine CCL21, and suggest that tissue stiffness may influence the ability of mDCs to reach lymph vessels or lymph nodes.

## Discussion

Here, we show that substrate stiffness influences specific aspects of differentiation and maturation of moDCs. In iDCs, CLR expression and CLR-mediated antigen internalization is decreased on 12 kPa, as well as β_2_ integrin cell surface expression and podosome formation. In mDCs, expression of co-stimulatory molecule CD83 and chemokine receptor CCR7 are significantly lower on 12 kPa, the latter resulting in lower chemokine-directed migration. This is the first study to report the effects of substrate stiffness on DC function and, together, our results clearly indicate that stiffness values of 12 kPa significantly inhibit several key functions of DCs during differentiation and maturation.

We show that MMR and DC-SIGN expression as well as MMR-dependent internalization of antigens by DCs is regulated by substrate stiffness. Interestingly, a recent study by Kianoush *et al*. demonstrated that MMR expression in RAW 264.7 macrophages is altered by surface topography^33^. Together, these data strongly suggest that CLR expression is regulated by a variety of mechanical cues. Remarkably, we also find that MMR expression is not rescued by adding more IL-4 and that exposure to 12 kPa substrates from day 3 in differentiation decreases MMR expression, despite an initial increase from day 0 to 3 under standard culture conditions. Thus, substrate stiffness appears to be a major determinant in C-type lectin mediated antigen uptake by iDCs and it would therefore be very interesting to study the role of tissue stiffness in orchestrating immune response to pathogens that contain many sugar moieties such as *Candida albicans* ^34^.

The fact that we do not observe differences in β_1_ expression for iDCs is interesting, since several studies show that the expression of β_1_ integrins or its associated α subunits is directly correlated with substrate stiffness in for example NIH 3T3 fibroblasts^18^, mouse mammary epithelial cells^35^ or B16F10 mouse melanoma cells^36^. Yet, these cell types often undergo major adaptations in cell morphology and adhesive behaviour related to the changes in substrate stiffness. This is in sharp contrast with the results presented in this study on iDCs where no stiffness-dependent changes in morphology were detected, which could explain the lack of a difference in morphology-related effects such as β_1_ integrin expression. Interestingly, we show that substrate stiffness did influence the expression of the leukocyte-restricted β_2_ integrins, which are essential for key functions of iDCs such as the formation of podosomes^32^. Indeed, we also show an altered ability of iDCs to form podosomes depending on substrate stiffness. The fact that β_2_ integrin expression is regulated by substrate stiffness may therefore have large consequences for iDC functionality in tissues that are stiffened due to fibrosis^19^ or tumour development^20^.

Although we observed an effect of substrate stiffness on the expression of the co-stimulatory molecule CD83, we did not observe an effect on the expression of CD86 and MHC-II as well as the ability of mDCs to stimulate T cells. Interestingly, studies investigating the effect of cyclic stretch or extracellular pressure did find altered expression levels of mDC markers as well as an altered ability of mDCs to stimulate T cells. One study subjecting murine DCs to cyclic stretch of 3% for 1 hour observed increased expression of all maturation markers tested (MHC-II, CD86, CD40)^37^, which was accompanied by an increased ability to stimulate T cells. In another study, increasing extracellular pressure with 40 mm Hg for 12 hours on human immature moDCs resulted in DCs with increased maturation markers (CD80, CD86, MHC-II), increased production of cytokines IL-12p70, IL-6, TNF-α and IFN-γ and increased CD4^+^ T cell proliferation^38^. Altogether, it thus seems that substrate stiffness is less determinant for the ability of mDCs to stimulate T cells compared to other types of mechanical cues. This is particularly interesting since DCs have been shown to change the deformability of lymph nodes^39^, the primary site of T cell stimulation, which would, according to our results, not affect their capacity to stimulate T cells.

We found that the expression of MMR, DC-SIGN, β_2_ integrins, CD83 and CCR7 is regulated by substrate stiffness. Interestingly, these proteins share transcriptional regulators that control their expression in myeloid cells. Expression of MMR, DC-SIGN and β_2_ integrins is regulated by the transcription factor PU.1^40–43^, a crucial player in DC development^44^ and the promoters of CD83 and CCR7 have been shown to be regulated by the transcription factor SP1^45,46^. Furthermore, it has recently been shown for DCs that the promoters of DC-SIGN and CD83 are demethylated by TET2 upon stimulation with IL-4^47^. We therefore hypothesize that the DC stiffness sensing pathways may converge to regulate the activity of these specific transcription factors or demethylases and that, as such, tissue stiffness plays a major role in regulating the phenotype and function of iDCs and mDCs.

To broadly investigate the effect of substrate stiffness on DCs, we aimed to use chemically equal substrates with stiffness values over a large range, encompassing the physiological range (0.5, 2 and 12 kPa) and the low to high patho-physiological range (12 and 50 kPa), two areas that partially overlap. Interestingly, we consistently observed that key functions during differentiation and maturation were inhibited in DCs conditioned on 12 kPa compared to DCs conditioned on 2 kPa, while the results on 50 kPa are ambiguous. DCs conditioned on 50 kPa displayed intermediate results compared to DCs 2 and 12 kPa (e.g. C-type lectin expression and ovalbumin uptake in iDCs), or comparable to 2 kPa (e.g. β_2_ integrins expression in iDCs and CCR7 expression in mDCs). Tissue stiffness typically increases during pathological conditions. During liver fibrosis, tissue stiffness increases from <6 kPa in healthy tissue to >12.5 kPa in stage 4 fibrosis^48^ and during lung fibrosis, tissue stiffness increases from ~2 kPa in healthy tissue to ~17 kPa in fibrotic lungs^49^. Furthermore, it is well known that cancer development is associated with tissue stiffening^50^. Tissue stiffness of 12 kPa or greater is referred to by Janmey and Miller as potentially leading to aberrant cell –cycle progression and abnormal tissue^51^. Based on our results, it is therefore plausible that pathological tissue stiffening *in vivo*, resulting in a stiffness shift from soft (~2 kPa) towards stiff (~12 kPa), can directly inhibit DC function by mechanically acting on these cells. Although less common, tissue stiffness can further increase to 50 kPa in for example liver cirrhosis^52^ or wound healing and scar tissue formation^53^. Based on our results, it is hard to predict how DCs would respond to such changes in the tissue microenvironment but it would be interesting to further dissect the effects of high stiffness values on DC function in the future. Our data clearly indicate that DC function is affected in the high stiffness range, something which could for example also be valuable in controlling the immunogenicity of tissue scaffolds or implants.

In summary, we for the first time provide evidence that a broad set of DC functions is regulated by substrate stiffness. Our data strongly suggest that DCs conditioned on stiff substrates (12 kPa), resembling pathologically stiffened tissue, perform suboptimal compared to DCs conditioned on soft substrates (2 kPa), resembling healthy tissue. DCs in stiffened tissue may therefore be hampered in evoking an immune response, critically affecting the clearance of pathogens or aberrant cells. This impairment not only holds implications for patho-physiological conditions, but also for non-physiological conditions such as the presence of implants or scaffolds. Stiffness of scaffolds may critically affect differentiation and activation of infiltrating immune cells and may determine whether they are immunogenic or not. The same holds true for implants, which stiffness may direct differentiation of interacting immune cells towards immunity or tolerance. In this study, we used 2D flat substrates fabricated from PAA to study the role of substrate stiffness in regulating DC phenotype and functionality. Future studies using biocompatible scaffolds with tuneable stiffness and minimal changes in ligand density and pore size should provide more insights whether the effects observed here also hold true in settings that better mimic the *in vivo* situation. Furthermore, efforts should be directed in providing mechanistic insight into the stiffness sensing pathways that control DC phenotype and function to control immune activation.

## Methods

### Cell isolation and culture on substrates with varying stiffness

DCs were generated from human peripheral blood mononuclear cells (PBMCs) as described previously^54^. Buffy coats of healthy individuals were purchased at Sanquin blood bank, Nijmegen, the Netherlands and PBMCs were isolated by density gradient centrifugation using Lymphoprep (Axis-Shield). From the PBMC fraction, monocytes were isolated by magnetic-activated cell sorting (MACS) using CD14 microbeads (Miltenyi Biotec). Isolated monocytes were cultured for 6 days at a density of 5*10^5^ cells per ml in X-VIVO15 medium supplemented with gentamicin (Lonza) and 2% human serum (Sanquin) in a 37 °C humidified and 5% CO_2_ containing atmosphere. Differentiation to DCs was induced by addition of 300 U/ml IL-4 and 450 U/ml GM-CSF (both from Cellgenix).

Cells were cultured in 6-well culture plates, either plastic polystyrene for standard tissue culture (Corning Costar) or 6-well easy coat Softwell plates, containing polyacrylamide (PAA) substrates with a stiffness of 0.5, 2, 12 or 50 kPa (Matrigen).

Prior to cell seeding, culture plates were by default coated with medium supplemented with 2% human serum. Additionally, for experiments presented in Supplementary Fig. S4, culture plates were coated with human fibronectin (FN) (2 µg/ml or 20 µg/ml, FN from human plasma, Sigma-Aldrich) or with a combination of FN followed by medium supplemented with 2% human serum for 30 minutes at 37 °C.

For late differentiation experiments, immature DCs were differentiated from day 0 to day 3 in T75 standard tissue culture plastic flasks (Corning Costar), generated from the monocytic fraction of PBMCs adhered to plastic. On day 3, cells were transferred to and cultured for 3 additional days on either standard 6-well plastic plates (Corning Costar) or 12 kPa 6-well easy coat Softwell plates in the presence of either the standard concentration of IL-4 (300 U/ml) or a concentration twice as high (600 U/ml).

To induce maturation, culture medium of DCs conditioned for 4 or 5 days in the presence of IL-4 and GM-CSF was supplemented with 5 ng/ml IL-1β, 10 ng/ml TNF-α, 15 ng/ml IL-6 and 10 µg/ml PGE_2_ and DCs were cultured for 2 more days before harvesting and subsequent analysis.

### Measurements of cell viability and cell surface marker expression

Differentiation and maturation marker expression was measured using flow cytometry. For differentiation markers the following antibodies were used: mouse-anti human CD206 (MMR; Clone 19.2) and mouse anti-human CD209 (DC-SIGN; Clone DCN46), both from BD Pharmingen; mouse anti-human CD14 (clone RM052; Immunotech), mouse anti-human β_2_ integrins (clone L19; from hybridoma) and rat anti-human β_1_ integrins (clone AIIB2; from hybridoma), and mouse anti-human CD68 (clone KP1; eBioscience). For maturation markers the following antibodies were used: mouse anti-human CCR7 (clone 150503, R&D Systems), mouse anti-human MHC-II (HLA-DR/DP; clone q5/13, from hybridoma), mouse anti-human CD83-FITC (clone HB15e) and mouse anti-human CD86-PE (clone 2331), both BD. Appropriate isotype controls were included in all measurements.

Goat-anti-mouse IgG(H+L)-Alexa488 or goat-anti-rat IgG(H+L)-Alexa488 secondary antibodies were used (Life Technologies). Cell viability was monitored with Fixable Viability Dye eFluor780 (eBioscience). Samples were measured on a CyAn ADP flow cytometer (Beckman Coulter). Analysis was performed with Flowjo Software (Treestar Inc) version 9.7.6. Cell surface marker geometric mean fluorescence intensities (gMFI) were obtained from the eFluor780-negative cell fraction and corrected with geometric mean fluorescence intensities measured for isotype controls.

### Western Blot

Whole cell protein lysates of day 6 immature DCs of 3 independent donors were prepared in protein lysis buffer (1% SDS, 0.04 M Tris pH 6.8 including proteinase and phosphatase inhibitors). Total protein concentration was measured using the Pierce BCA Protein Assay Kit (23225, Thermo Scientific). 40 µg of total protein was run on 8% SDS-PAGE gels and proteins were transferred to PVDF membranes (Immobilon-FL) by wet electrophoretic transfer. Membranes were blocked for 1 hour at RT in TBS-T(0.1% Tween) with 5% non-fat dry milk and incubated overnight in TBS-T with 5% non-fat dry milk with primary antibodies: rabbit polyclonal anti-human MMR (#12981, Cell Signalling Technology) or rabbit polyclonal anti-human DC-SIGN (clone H200; Santa Cruz Biotechnology). β tubulin, indicated with mouse monoclonal anti-human β tubulin antibody (Clone E7; from hybridoma) was used as loading control. Membranes were incubated with secondary antibodies (goat-anti-rabbit-IRDye680 and goat-anti-mouse IRDye800, both from LI-COR) for 1 hour at RT in TBS-T with 5% non-fat dry milk and subsequently scanned using an Odyssey-CLx imaging system (LI-COR). Resulting images were analysed with FIJI ImageJ. Integrated intensity of protein bands was corrected for integrated intensity of the background. Resulting integrated intensity of protein of interest was corrected for protein loading controls and normalized to results of 2 kPa conditions.

### Ovalbumin and transferrin internalization assays

For ovalbumin internalization, 1*10^5^ immature moDCs were incubated with 10 µg/ml Ovalbumin-Alexa488 (Invitrogen) in serum-free medium for 10, 20, 30 or 60 minutes at 37 °C and 5% CO_2_. Control samples were incubated for 60 minutes at 4 °C (no internalization). For transferrin internalization, 1*10^5^ immature moDCs were incubated with 5 µg/ml biotinylated human transferrin (Sigma) at 4 °C in serum-free medium. After washing steps, samples were incubated at 37 °C and 5% CO_2_ for 5, 10, 20 or 30 minutes. Control samples were incubated for 30 minutes at 4 °C (no internalization). Finally cells were incubated with streptavidin-Alexa488 (Thermo Fischer Scientific) at 4 °C. All samples were measured on a CyAn ADP flow cytometer (Beckman Coulter). Internalization of ovalbumin-Alexa488 is represented as geometric mean of fluorescence intensity. The percentage of internalized transferrin was calculated by dividing the difference of the gMFI of the control sample minus the gMFI of the time point, by the gMFI of the control sample, multiplied by 100%.

### Microscopy

Brightfield images of iDCs and mDCs in cell culture were acquired with a Leica-DMI6000 widefield microscope using a Leica 40 × 0.55 NA Dry objective.

To evaluate cell velocity of mDCs on their culture substrate, brightfield time-lapse imaging of mDC culture plates at 37 °C and 5% CO_2_ was performed using a Zeiss Axiovert 200 M with Moticam-pro 2850 CCD Camera, Okolab stage incubator and run by Micromanager 1.4 software, using an Olympus 20 × 0.4 NA dry objective. Brightfield images were taken every minute for 1 hour.

To check the effect of coating protein concentration on effective coating of the substrates, PAA substrates were incubated with 2 µg/ml or 20 µg/ml in PBS for 30 minutes at 37 °C and 5% CO_2_. Subsequently, substrates were incubated with anti-human fibronectin (rabbit polyclonal IgG – F3648 – Sigma Aldrich) followed by secondary antibody goat-anti-rabbit-Alexa488 (Life Technologies). Coatings were imaged using a Leica DMI-6000 widefield microscope with a Leica 10 × 0.30 NA Dry objective.

To evaluate podosome formation, immature moDCs were seeded onto #1.5 12 mm glass coverslips (Electron Microscopy Sciences) coated with human fibronectin (Sigma) and left to adhere for 3 hours at 37 °C and 5% CO_2_. Subsequently, cells were fixed with 4% paraformaldehyde. Samples were permeabilized with 0.1% Triton-X-100 (Sigma) in PBS, then blocked with a 2% BSA solution and incubated with primary mouse-anti-human vinculin antibody (clone hVIN-1, Sigma) and secondary goat-anti-mouse IgG(H+L)-Alexa488 antibody (Life Technologies). Finally samples were stained with DAPI (Sigma) and Phalloidin-Alexa633 (Life Technologies) to stain the nucleus and the actin cytoskeleton respectively. Coverslips were embedded in Mowiol (Sigma). Samples were examined using a Leica DMI-6000 widefield microscope with a Leica 63 × 1.40 NA oil immersion objective.

To determine ovalbumin internalization, immature moDCs from 60 min Ovalbumin internalization samples were fixed in suspension using 4% paraformaldehyde. Cells were left to settle on #1.5 12 mm glass coverslips (Electron Microscopy Sciences) coated with Poly-L-lysine and again fixed with 4% paraformaldehyde. Subsequently, cells were blocked with 2% BSA and incubated with primary mouse anti-human MHC-II (HLA-DR/DP) (see above) and secondary Goat-anti-Mouse (H+L)-Alexa647 (Life Technologies). Coverslips were embedded in Mowiol. Samples were imaged on an Olympus FV1000 confocal laser scanning microscope with a 60 × 1.35 NA oil immersion objective.

All images were analyzed using FIJI ImageJ Software version 1.50.i. Cell velocity of mDCs was determined (in 2 donors) by manually tracking in total >30 single cells per condition over a 1 hour time period in brightfield images using the Manual Tracking plugin in FIJI ImageJ. Fibronectin coating intensity was determined by averaging the mean gray value of 4-5 fields of view per condition, measured in FIJI ImageJ. Cell clump size was determined in brightfield images (n = 2 donors) by averaging the area of >40 cell clumps in >8 fields of view per condition, measured in FIJI ImageJ.

### Transwell migration assay

For transwell migration, mature moDCs were first detached from the PAA substrates. Subsequently, 1*10^5^ mature moDCs were added to 6.5 µm pore size transwells (Corning Costar) and left to transmigrate for 2.5 hours at 37 °C and 5% CO_2_ towards the bottom compartment containing X-VIVO15 medium with 2% human serum with or without 100 ng/ml Recombinant human CCL-21 (6Ckine carrier-free, Biolegend). Input control samples were taken along. After 2.5 hours, all samples were collected in equal volumes and cell numbers in these suspensions were counted automatically by analyzing a fixed volume using the MACSQuant flow cytometer (Miltenyi Biotec). Analysis was performed with Flowjo Software (Treestar Inc) version 9.7.6. Experiments were performed in duplo.

### T cell proliferation assay

Mature moDCs were co-cultured with allogeneic peripheral blood lymphocytes (PBL) at a ratio of 1:10 in X-VIVO15 medium with 2% serum at 37 °C and 5% CO_2_. As a negative control PBLs only were taken along, as a positive control PBLs were incubated with 4:1 human T-activator CD3/CD28 Dynabeads (Invitrogen). After 4 days of culture the proliferative response of the PBLs was determined by [^3^H]-thymidine incorporation (MP Biomedicals). The incorporated [^3^H]-thymidine was measured after 8 hours by liquid scintillation spectroscopy. Experiments were performed in triplo.

### Statistical analysis

Statistical analysis was performed using Graphpad Prism version 5.03 (Graphpad Software). Statistical significance was tested using 2-tailed paired t-test for comparison of 2 conditions; repeated measures ANOVA was performed for comparison of 3 or more conditions, using Tukey’s range test for post-hoc analysis. For comparison of cell clump size results, one-way ANOVA was used. The number of donors reflects the number of independent data points for each experiment. For flow cytometry experiments, at least 2000 cells were analysed per sample. Differences were considered statistically significant at p < 0.05.

### Data availability

All data are available from the authors upon request.

## Electronic supplementary material

Supplementary Information
