# Supplementary material for: Substrate stiffness influences phenotype and function of human antigen-presenting dendritic cells
Source: Sci Rep. 2017 Dec 13;7:17511. doi: 10.1038/s41598-017-17787-z (PMC5727489; doi:10.1038/s41598-017-17787-z)
Supplement: Supplementary file 1 — Supplementary Information [file 41598_2017_17787_MOESM1_ESM.pdf]

Supplementary Information

Substrate stiffness influences phenotype and function of human antigen-presenting dendritic cells

Authors:

Svenja F.B. Mennens[1], Matteo Bolomini-Vittori[1], Jorieke Weiden[2], Ben Joosten[1], Alessandra Cambi[1]* and Koen van den Dries[1]*

1. Department of Cell Biology
Radboud Institute for Molecular Life Sciences
Radboud University Medical Center
Geert Grooteplein Zuid 26-28
6525 GA Nijmegen
The Netherlands

2. Department of Tumor Immunology
Radboud Institute for Molecular Life Sciences
Radboud University Medical Center
Geert Grooteplein Zuid 26-28
6525 GA Nijmegen
The Netherlands

*Corresponding authors

Figure S1

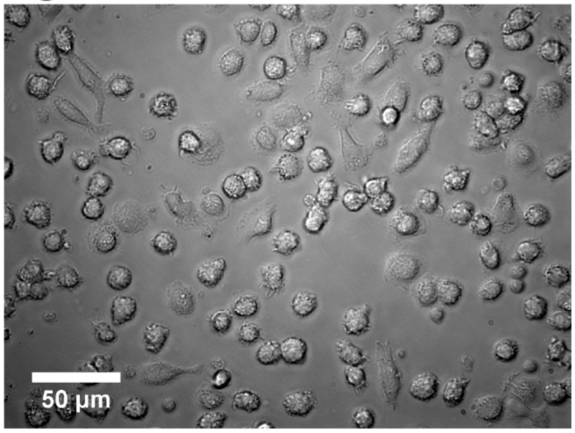

Supplementary Figure S1. Morphology of day 3 iDCs on tissue culture plastic
Representative brightfield images taken with a 40x objective of iDCs in culture on standard tissue culture plastic after 3 days of differentiation.

Figure S2

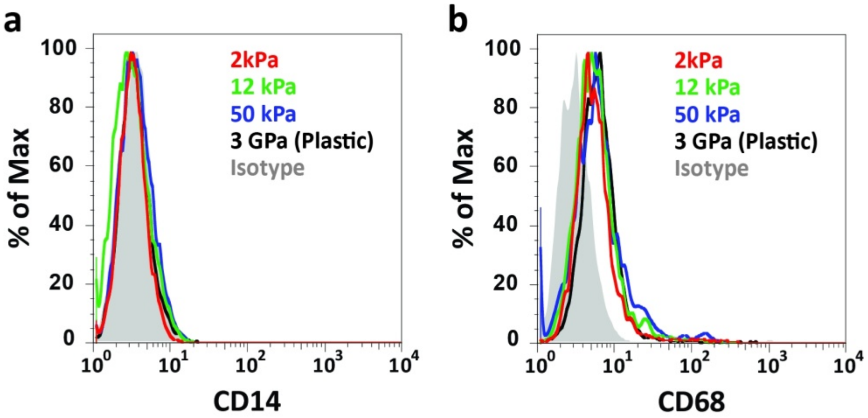

Supplementary Figure S2. iDCs conditioned on PAA surfaces display a DC-like phenotype
Fluorescence intensity profiles of CD14 expression (**a**, representative of n = 5 donors) and CD68 expression (**b**, n = 1 donor) in iDCs conditioned for 6 days in the presence of IL-4 and GM-CSF on PAA surfaces of 2, 12, 50 kPa and standard tissue culture plastic measured with flow cytometry.

Figure S3

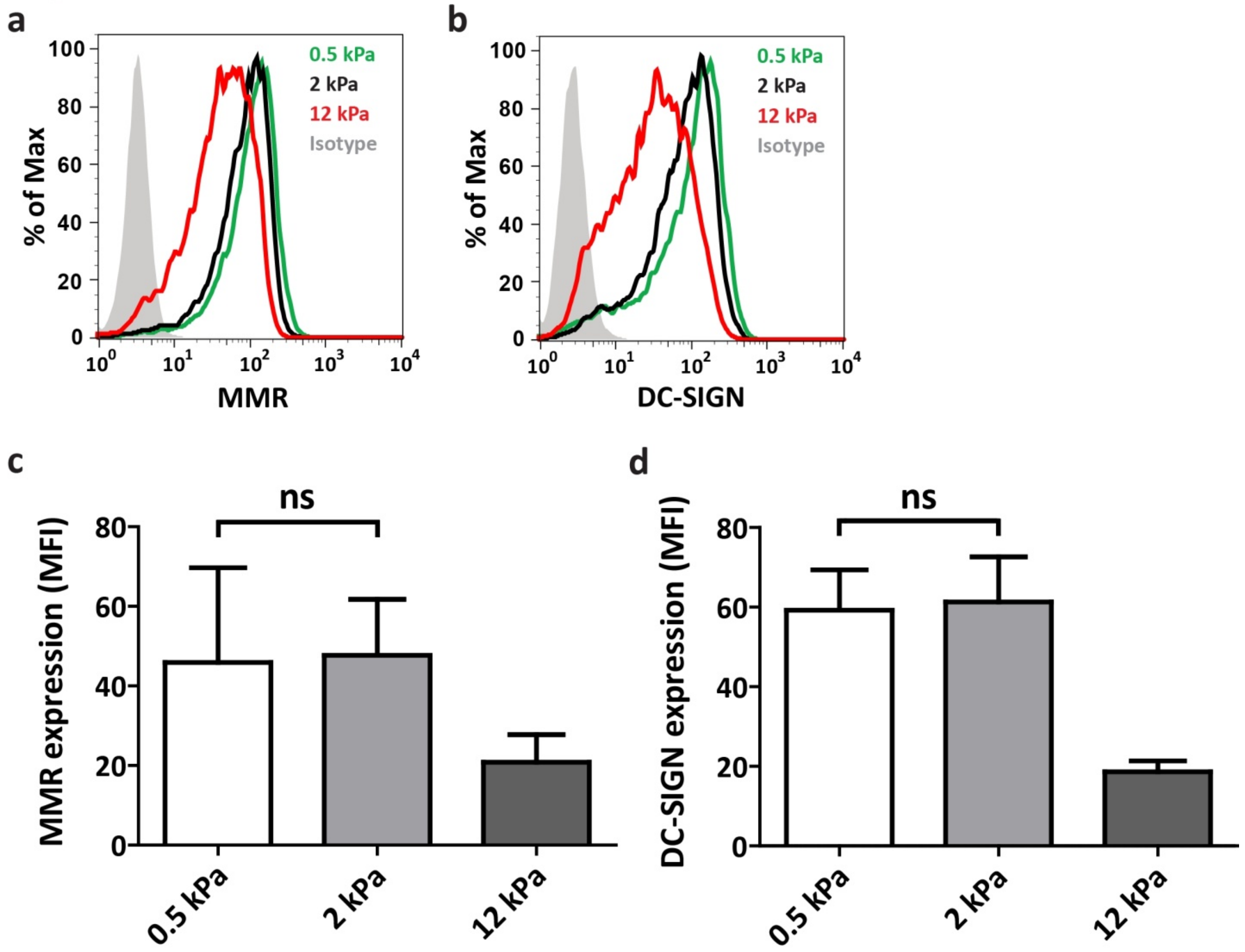

Supplementary Figure S3. Conditioning on PAA substrates softer than 2 kPa yields C-type lectin expression levels comparable to 2 kPa.

a,b. Representative fluorescence intensity profiles of MMR-expression (**a**) and DC-SIGN expression (**b**) in iDCs conditioned for 6 days on PAA surfaces of 0.5 (green line), 2 (black line) and 12 kPa (red line).

c,d. Cell surface expression of C-type lectins MMR (**c**) and DC-SIGN (**d**) (both n = 3 donors) on iDCs conditioned for 6 days on PAA surfaces of 2, 12 and 50 kPa, represented as geometric mean fluorescence intensity measured with flow cytometry. Bars represent mean with SEM. Statistical significance was tested with repeated measures ANOVA.

Figure S4

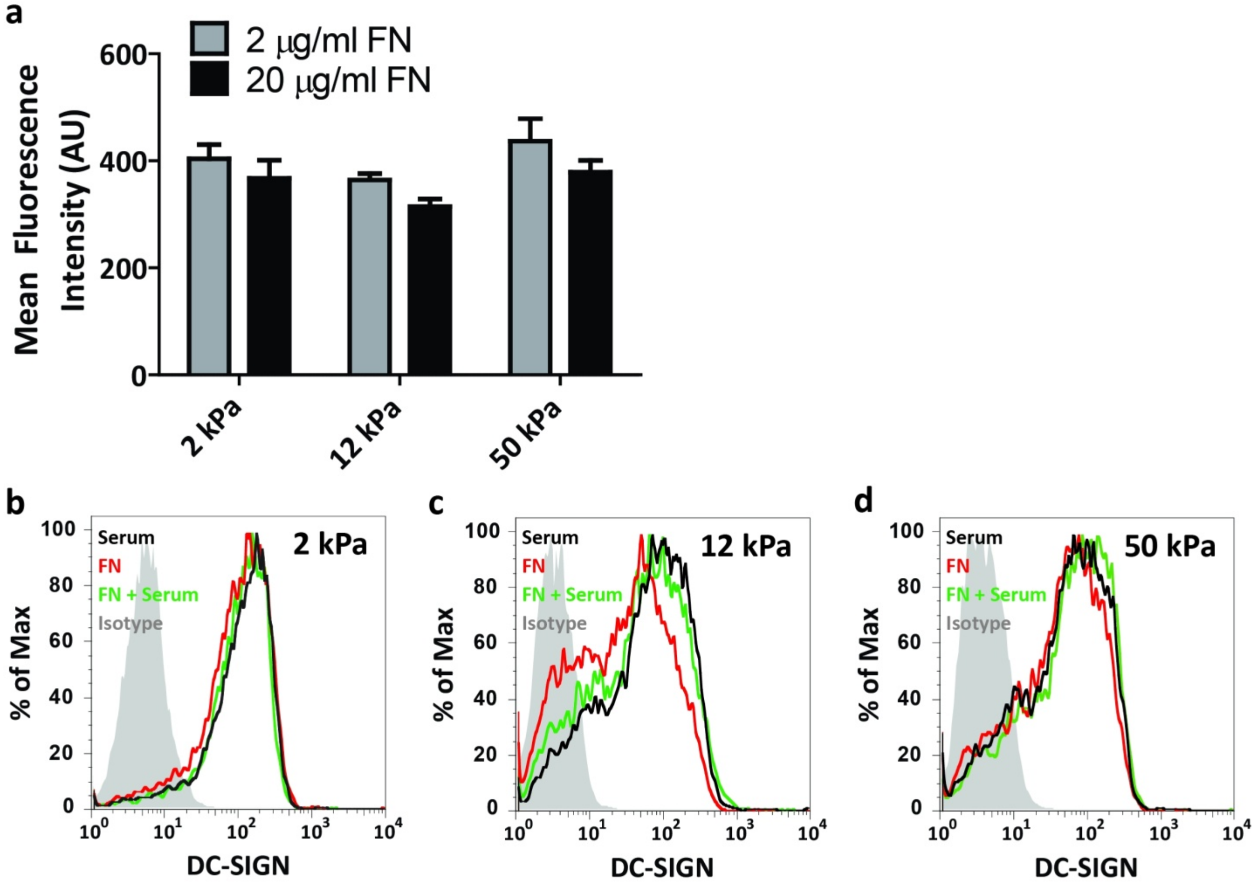

Supplementary Figure S4. CLR expression of iDCs on PAA surfaces is not influenced by ligand coating

a. Mean fluorescence intensity from PAA substrates coated with fibronectin (2 or 20 μg/ml) and labelled with anti-human fibronectin antibody. Average of >4 fields of view per condition.

b-d. Fluorescence intensity profiles of DC-SIGN expression (n = 1 donor) in iDCs conditioned for 6 days on PAA surfaces of (**b**) 2 kPa, (**c**) 12 kPa and (**d**) 50 kPa, coated with different coatings: serum (black line), fibronectin (FN: red line), or FN followed by serum (green line).

Figure S5

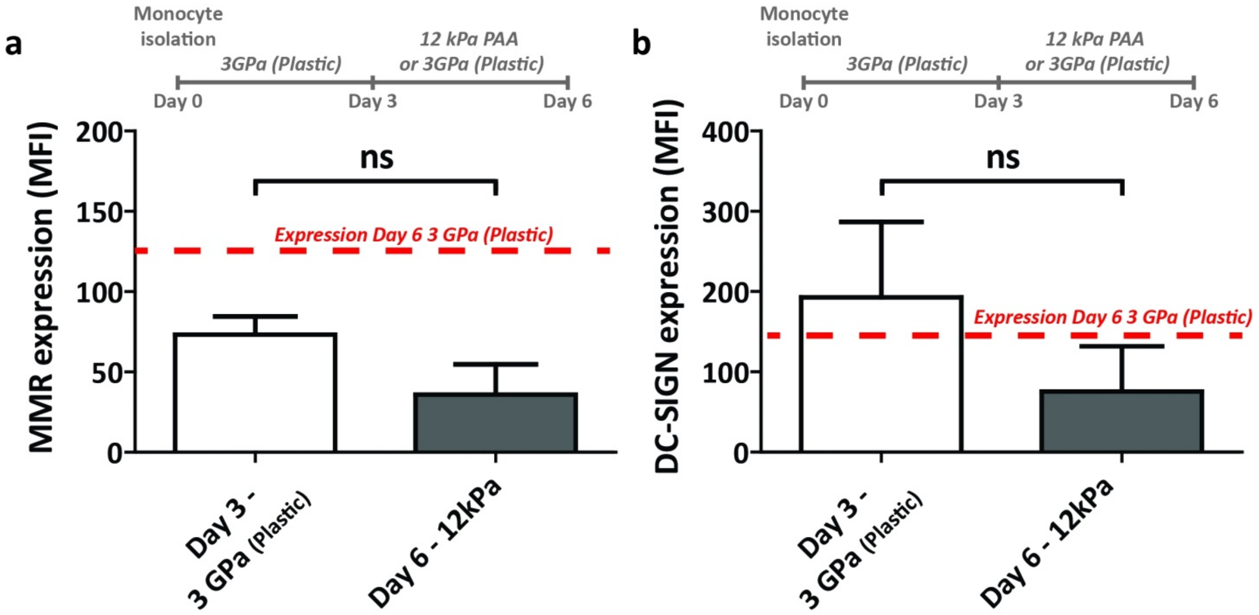

Supplementary Figure S5. Pre-existing expression of C-type lectins inhibited by transfer to 12 kPa PAA substrates.

Cell surface expression of MMR (**a**) and DC-SIGN (**b**) in iDCs (both n = 3 donors), cultured for 3 days on tissue culture plastic (day 3 – 3 GPa (Plastic)), and subsequently 3 more days on 12 kPa PAA surfaces (Day 6 – 12 kPa) or tissue culture plastic (mean fluorescence intensity indicated as red dashed line). Cell surface expression is represented as geometric mean fluorescence intensity measured with flow cytometry. Bars represent mean with SEM. Statistical significance was tested with 2-tailed paired t-test.

Figure S6

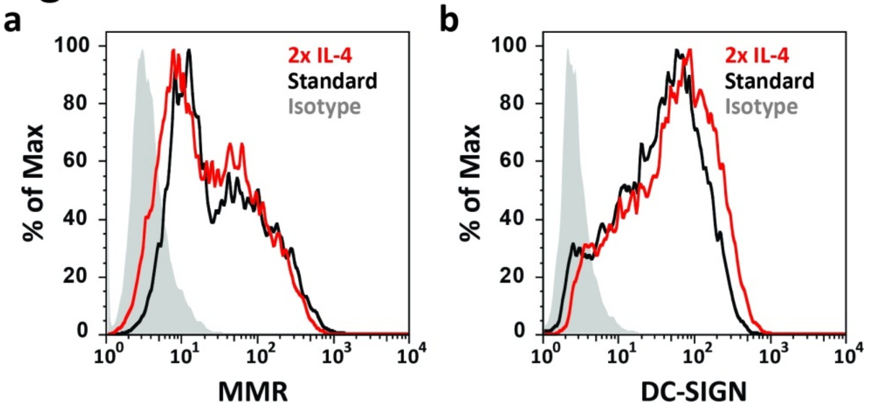

Supplementary Figure S6. IL-4 does not rescue the lower CLR expression of iDCs on 12 kPa
Fluorescence intensity profiles of cell surface MMR expression (**a**) and DC-SIGN expression (**b**) of iDCs differentiated from day 0 to day 3 in standard plastic culture flasks and from day 3 to day 6 on 12 kPa PAA surfaces in the presence of the standard (black line) or double (red line) concentration of IL-4 (n = 1 donor) in culture.

Figure S7

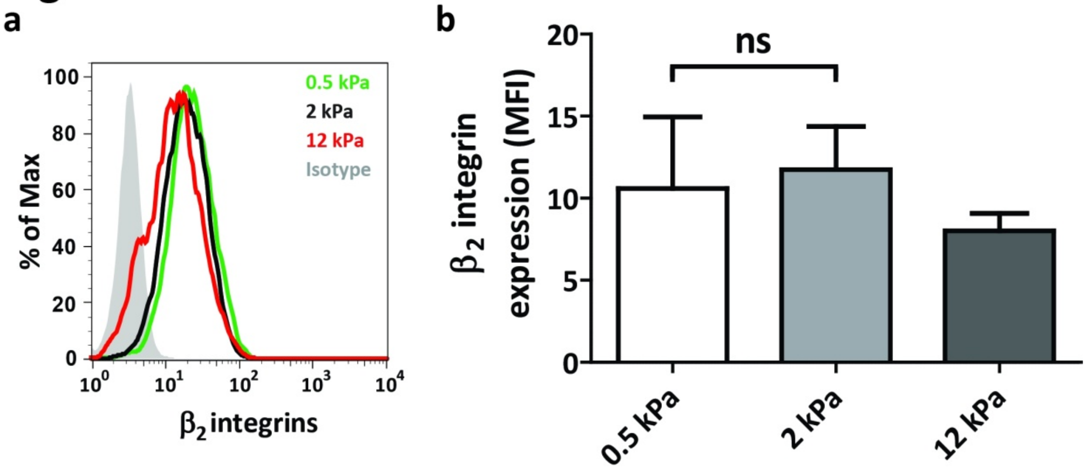

Supplementary Figure S7. Conditioning on PAA substrates softer than 2 kPa yields β_2 integrin expression levels comparable to 2 kPa.

a. Representative fluorescence intensity profiles of β_2 integrin expression in iDCs conditioned for 6 days on PAA surfaces of 0.5 (green line), 2 (black line) and 12 kPa (red line).

b. Cell surface expression of β_2 integrins (n = 3 donors) on iDCs conditioned for 6 days on PAA surfaces of 2, 12 and 50 kPa, represented as geometric mean fluorescence intensity measured with flow cytometry. Bars represent mean with SEM. Statistical significance was tested with repeated measures ANOVA.

Figure S8

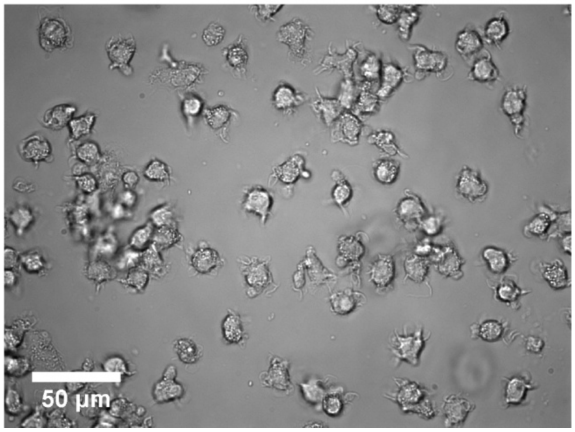

Supplementary Figure S8. Morphology of day 1 mDCs on tissue culture plastic

Representative brightfield images taken with a 40x objective of mDCs in culture on standard tissue culture plastic after 1 day of maturation (total 5 days of culture).

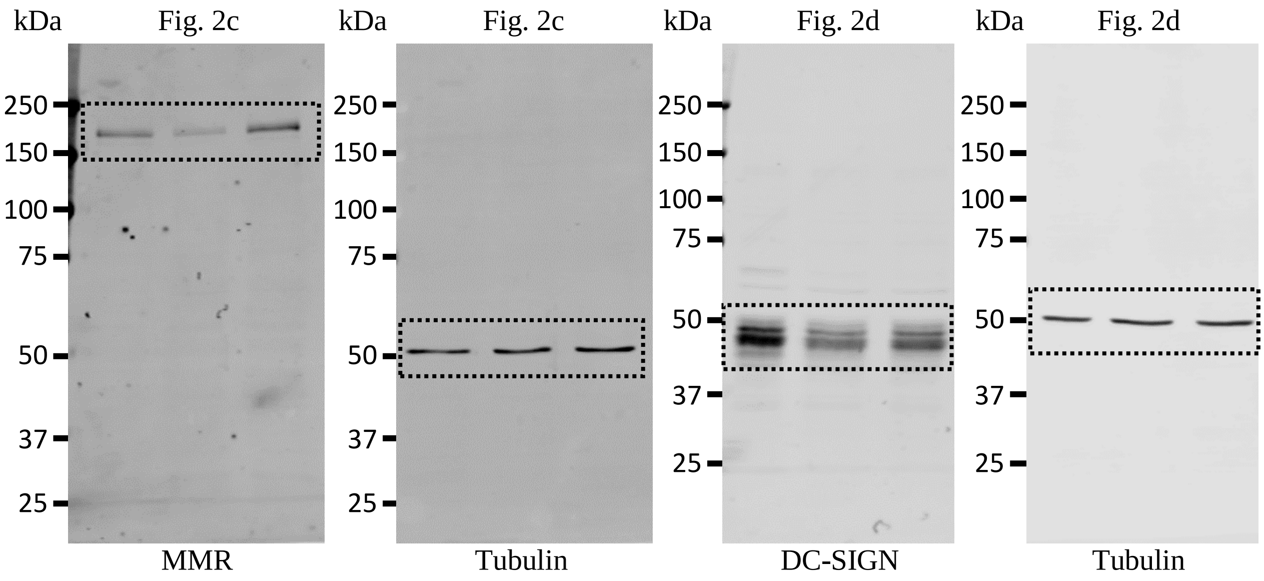

Supplementary Figure S9. Unprocessed scans of immunoblots
The figure panel, the name of the protein labelled, and molecular weight markers (in kDa) are indicated.
